# Novel mutations identified in Chinese families with autosomal dominant congenital cataracts by targeted next-generation sequencing

**DOI:** 10.1186/s12881-019-0933-5

**Published:** 2019-12-16

**Authors:** Shan Li, Jianfei Zhang, Yixuan Cao, Yi You, Xiuli Zhao

**Affiliations:** 1 Department of Medical Genetics, Institute of Basic Medical Sciences Chinese Academy of Medical Sciences - School of Basic Medicine Peking Union Medical College, 5 Dong Dan San Tiao, Dongcheng District, Beijing, 100005 People’s Republic of China; 2 The No.4 hospital (eye hospital) of Zhangjiakou, Zhangjiakou, 075000 People’s Republic of China

**Keywords:** Congenital cataract, Next-generation sequencing, Gene mutation, Bioinformatics analysis

## Abstract

**Background:**

Congenital cataract is a clinically and genetically heterogeneous visual impairment. The aim of this study was to identify causative mutations in five unrelated Chinese families diagnosed with congenital cataracts.

**Methods:**

Detailed family history and clinical data were collected, and ophthalmological examinations were performed using slit-lamp photography. Genomic DNA was extracted from peripheral blood of all available members. Thirty-eight genes associated with cataract were captured and sequenced in 5 typical nonsyndromic congenital cataract probands by targeted next-generation sequencing (NGS), and the results were confirmed by Sanger sequencing. Bioinformatics analysis was performed to predict the functional effect of mutant genes.

**Results:**

Results from the DNA sequencing revealed five potential causative mutations: c.154 T > C(p.F52 L) in *GJA8* of Family 1, c.1152_1153insG(p.S385Efs*83) in *GJA3* of Family 2, c.1804 G > C(p.G602R) in *BFSP1* of Family 3, c.1532C > T(p.T511 M) in *EPHA2* of Family 4 and c.356G > A(p.R119H) in *HSF4* of Family 5. These mutations co-segregated with all affected individuals in the families and were not found in unaffected family members nor in 50 controls. Bioinformatics analysis from several prediction tools supported the possible pathogenicity of these mutations.

**Conclusions:**

In this study, we identified five novel mutations (c.154 T > C in *GJA8*, c.1152_1153insG in *GJA3*, c.1804G > C in *BFSP1*, c.1532C > T in *EPHA2*, c.356G > A in *HSF4*) in five Chinese families with hereditary cataracts, respectively. NGS can be used as an effective tool for molecular diagnosis of genetically heterogeneous disorders such as congenital cataract, and the results can provide more effective clinical diagnosis and genetic counseling for the five families.

## Background

Congenital cataract is a clinically and genetically heterogeneous lens disorder, characterized by opacification of crystalin lens at birth or during early childhood [1]. The prevalence of congenital cataracts varies from 1 to 6 per 10,000 live births [2]. Approximately one third of the cases have a family history [3]. The cataract may be an isolated anomaly, or part of a multisystem syndrome [4]. Both X-linked and autosomal recessive inheritance patterns have been reported for congenital cataract, however autosomal dominant trait is the most prevalent mode [5–7]. Cataracts can be classified as sutural, whole lens, nuclear, lamellar, cortical, polar, cerulean, coralliform, and other subtypes, according to morphology of lens [8–10].

To date, at least 30 pathogenic genes have been found to link to congenital cataracts. From the reported mutant genes in congenital cataract families, nearly half of the mutations associated with crystalin genes [11], including genes coding for crystalin families (*CRYAA*, OMIM 604219; *CRYAB*, OMIM 613763; *CRYBA1, OMIM* 600881; *CRYBB1,* OMIM 611544; *CRYBB2,* OMIM 601547; *CRYBB3,* OMIM 609741; *CRYGC,* OMIM 604307; *CRYGD,* OMIM 115700; *CRYGS,* OMIM 116100), gap junctional proteins (*GJA3,* OMIM 601885; *GJA8,* OMIM 116200), beaded filament structural proteins (*BFSP1,* OMIM 611391; *BFSP2,* OMIM 611597), and other functional genes (e.g., *HSF4,* OMIM 116800; *MIP,* OMIM 615274; *PITX3,* OMIM 610623; *EPHA2*, OMIM 116600) [7, 9, 12–15].

Identification of accurate genetic cause of congenital cataract is essential for providing precise diagnosis and genetic counseling [8]. However, due to the high clinical and genetic heterogeneities, clinical and genetic diagnostic of congenital cataract, especially for nonsyndromic congenital cataracts, are limited by the traditional sequencing method, by which only few candidate genes can be sequenced at each time [16]. Recently, the next generation sequencing (NGS) combined with targeted genomic enrichment has proved to be an effective solution to the genetic test of genetically heterogeneous diseases and provides a new opportunity for genetic diagnostics of congenital cataracts [12, 17].

In this study, we collected information from five large Chinese families with congenital cataracts. Then we performed targeted enrichment and deep sequencing to detect the genetic mutations in these families. We identified five novel mutations in the *GJA3* (S385Efs*83), *GJA8* (F52 L), *BFSP1* (G602R), *EPHA2* (T511 M) and *HSF4* (R119H) genes that potentially resulted in the development of congenital cataract. With Sanger sequencing, we confirmed that mutations were co-segregated with affected individuals in the five families, whereas mutations were not found in unaffected family members and normal controls. Bioinformatics analysis, conservative prediction and 3-D protein simulation indicated that the five mutations might be the pathogenic mutations for congenital cataract families. This study demonstrates that the targeted gene sequencing can be used as an effective tool for genetics diagnosis of congenital cataract.

## Materials and methods

### Clinical examination and isolation of genomic DNA

Five Chinese pedigrees with autosomal dominant hereditary cataract were collected from The No.4 hospital (eye hospital) of Zhangjiakou, Hebei, China, and 50 unrelated subjects without eye diseases were enrolled as normal controls. Informed written consents were obtained from all adult participants and the legal guardians of children under age 18 and 3–5 mL peripheral blood samples were collected from all available members. Affected individuals were confirmed by histories of cataract surgery or ophthalmological examinations, and their clinical phenotypes were recorded by slit-lamp photography. Genomic DNA was extracted from peripheral blood using standard SDS-proteinase K-phenol/chloroform method [18]. This study was approved by the Institutional Review Board (IRB) of the Institute of Basic Medical Sciences, Chinese Academy of Medical Sciences, Beijing, China (015–2015).

### Targeted capturing and next generation sequencing

A capture array (NimbleGen, Roche) was designed to capture all exons, splice sites and adjacent introns sequences of 38 known pathogenic genes associated with inherited cataract diseases based on GeneReviews (NCBI) [12, 19] (Additional file 1: Table S1). Genomic DNA was fragmented ranging from 200 bp to 250 bp and purified, followed by treatment with T4 DNA polymerase, T4 phosphonucleotide kinase and Klenow fragment of DNA polymerase to fill 5′ overhangs and to remove 3′ overhangs. According to standard Illumina protocols, terminal A residues were added following a brief incubation with the Klenow 3′-5′ exo-enzyme and dATP. Adapter oligonucleotides from Illumina (single reads) were ligated to the ends. Subsequently, ligation was confirmed by four-cycle PCR using a high-fidelity polymerase with primers containing a custom-synthesized barcode sequence (8 bp) as a sample index signature. PCR generated a library for further analysis, and the indexed fragments and DNA adapter-ligated were pooled and hybridized to the capture array. After hybridization and enrichment, the DNA sample was sequenced on Illumina HiSeq2000 Analyzers to generate paired end reads (90 bps) [17]. Raw data was generated by Illumina Pipeline, followed by imaging analysis and base calling. Short-reads mapping was then mapped to the human genome reference from the NCBI database (Build 37) using the Multi-Vision software package of Burrows Wheeler Aligner. Single nucleotide variants (SNVs) were determined by SOAPsnp, and small insertion and deletions (InDels) were identified using the GATK InDel Genotyper. Previously identified SNPs were determined using the NCBI dbSNP (http://www.ncbi.nlm.nih.gov/SNP/) or HapMap databases (http://hapmap.ncbi.nlm.nih.gov/). Known disease-causing mutations were identified from the Human Gene Mutation Database HGMD (http://www.hgmd.org/) or from mutations reported previously. All reference sequences were based on the NCBI37/hg19 assembly of the human genome.

### Sanger sequencing

To validate the DNA variants (substitutions or indels) generated from next-generation sequencing, the target sites and their flanking sequences were examined by PCR combined with Sanger DNA sequencing in the corresponding proband. Genomic DNA reference sequences of *GJA8* (NM_005267.4), *GJA3* (NM_021954.3), *BFSP1* (NM_001195.3), *EPHA2* (NM_004431.3) and *HSF4* (NM_001538.3) were obtained from the University of California, Santa Cruz (UCSC) Genome Browser database (http://genome.ucsc.edu/). All primers for PCR were designed via online tool Primer3 (http://primer3.ut.ee/) (Table 1). Sanger sequencing was then performed in all probands and unaffected family members. The PCR program was performed as following: 95 °C for 3 min; 94 °C for 30 s, 58 °C for 30 s, 72 °C for 40 s (38 cycles); 72 °C for 8 min. The PCR products were separated by 2% agarose gel electrophoresis, and the target fragment was purified by the QIAquick Gel Extraction kit (Qiagen, Hilden, Germany) according to the manufacturer’s protocol. Sequencing result from Applied Biosystems 3730xl DNA Analyzer (Thermo Fisher Scientific, Waltham, MA, USA) was aligned to reference sequence through CodonCode Aligner (version 6.0.2.6; CodonCode, Centerville, MA, USA).

**Table 1:** Primers for PCR and Sanger sequencing used in mutation validation

Gene	Transcript	mutation	Forward primer (5′-3′)	Reverse primer (5′-3′)	Product length (bp)
*GJA8*	NM_005267.4	c.154 T > C	GCAACTTGGAAAGGAGAGGT	ATGTGGCAGATGTAGGTCCT	576
*GJA3*	NM_021954.3	c.1152_1153insG	GATGACTGAGCAGAACTGGG	CCTGATCTCTCCTCCATCGT	496
*BFSP1*	NM_001195.3	c.1804G > C	CCAATTGACCAGCAGCCTAT	CTGTCCTCATGAAGCTGACC	623
*EPHA2*	NM_004431.3	c.1532C > T	AATTCCGAGCCTCAGTTTCC	TGAACTTCCTCACACCACTG	540
*HSF4*	NM_001538.3	c. 356G > A	CTGCCCCAGTATTTCAAGCA	CCTCCTCTTTGCTCATTCCC	309

### Bioinformatics analysis

The amino acid sequences of protein Gap junction alpha-8 protein (encoded by *GJA8*), Filensin (encoded by *BFSP1*), Ephrin type-A receptor 2 (encoded by *EPHA2*), and Heat shock factor protein 4 (encoded by *HSF4*) were obtained from NCBI Protein database (FASTA format). Multiple sequence alignments with different animals (humans, mouse, chicken, monkey, snake, frog and zebrafish) and conservative analysis were performed by the software MEGA (Version7; Institute for Genomics and Evolutionary Medicine, Temple University, USA). Functional effects of the mutations was predicted by Online tools Polymorphism Phenotyping version2 (PolyPhen-2; http://genetics.bwh.harvard.edu/pph2/), Scale-Invariant Feature Transform (SIFT; http://sift.jcvi.org/), Protein Variation Effect Analyzer (PROVEAN; http://provean.jcvi.org/seq_submit.php), Mutation Taster (http://www.mutationtaster.org) and M-CAP (http://bejerano.stanford.edu/mcap/) to assess the possible effects of variants on protein structure and function, regarding sequence conservation, chemical change, and likelihood of pathogenicity. 3D structures of normal and missense mutants in Gap junction alpha-8 protein (PDB: 6MHY), Ephrin type-A receptor 2 (PDB: 2X10) and Heat shock factor protein 4 (PDB: 2IDU) were generated by homology modeling using SWISS-MODEL (http://swissmodel.expasy.org/), however, because wild-type human filensin hadn’t been crystallized and there was no homologous sequence of filensin, a 3D model of filensin couldn’t be generated. The interactions between the amino acid and the neighboring residues were exhibited and simulated by PyMOL (Schrödinger, LLC, New York, NY, USA; http://www.pymol.org/).

## Results

### Clinical evaluation

#### Family 1

**Fig. 1:**
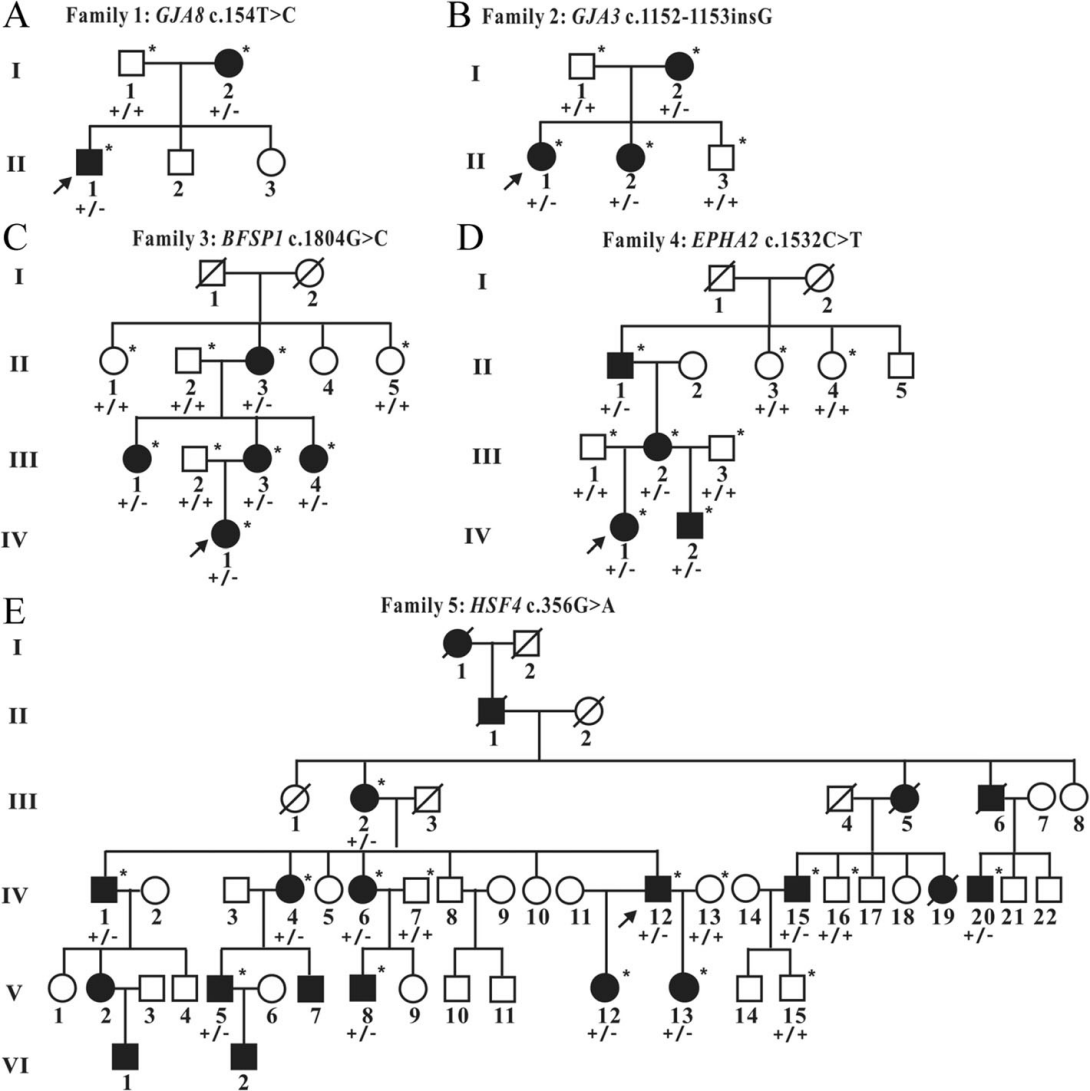
The pedigrees and genotypes of 5 Chinese families with congenital autosomal dominant cataracts. The probands are indicated with an arrow. Squares and circles symbolize male and female individuals respectively. Black symbols indicate affected members and blank symbols indicate unaffected individuals. Asterisks indicate sequenced samples. “+/+” indicates wild-type and “+/−” indicates heterozygote. (**a**) Pedigree of Family 1, all patients carried the heterozygous mutation c.154 T > C in *GJA8*. (**b**) Pedigree of Family 2, all patients carried the heterozygous mutation c.1152_1153insG in *GJA3*. (**c**) Pedigree of Family 3, all patients carried the heterozygous mutation c.1804G > C in *BFSP1*. (**d**) Pedigree of Family 4, all patients carried the heterozygous mutation c.1532C > T in *EPHA2*. (E) Pedigree of Family 5, all patients carried the heterozygous mutation c.356G > A in *HSF4*

**Fig. 2:**
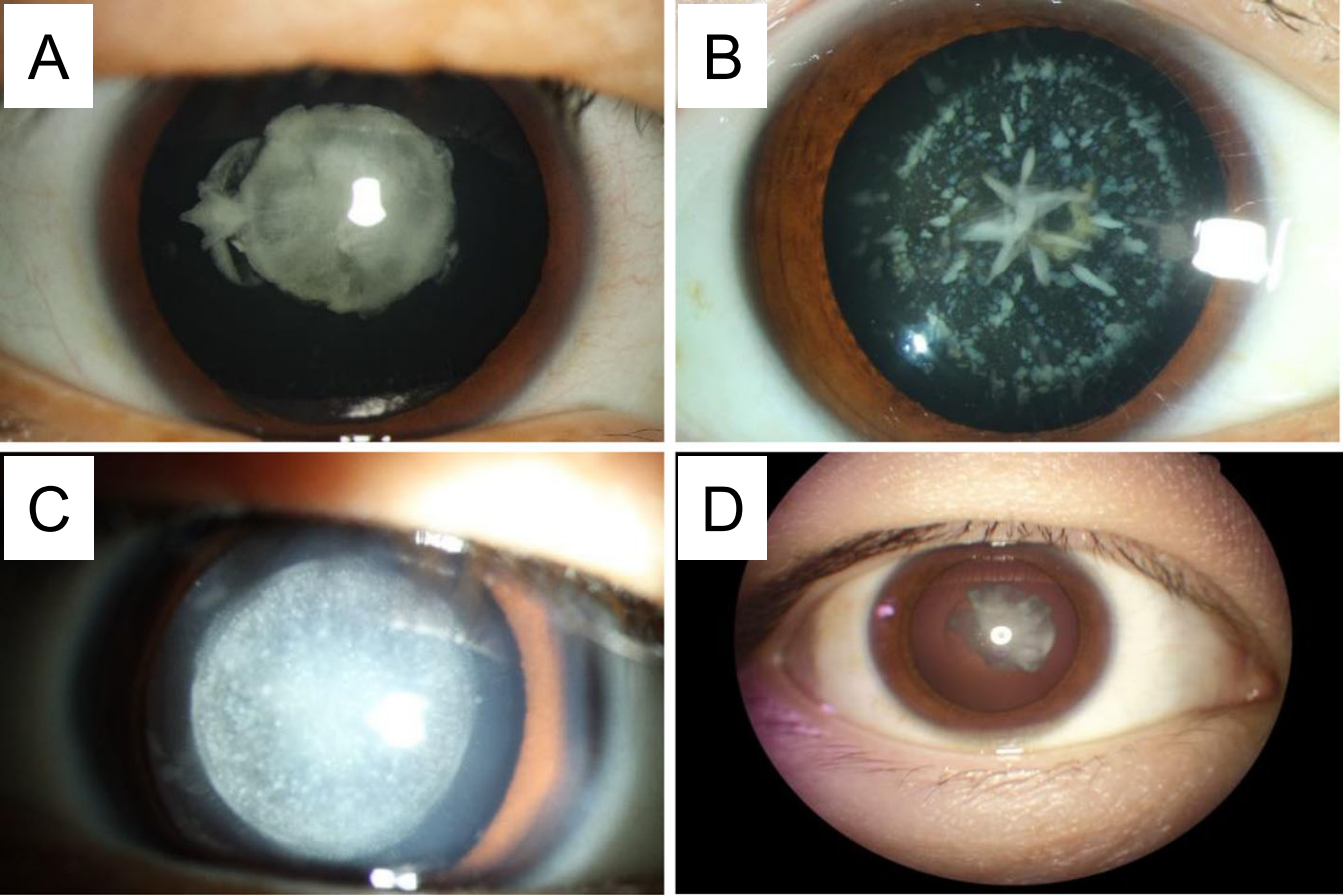
Slit lamp photographs of the patients from four families. (**a**) The proband (II1) in the Family 1 presented nuclear cataract. (**b**) The proband (II1) in Family 2 showed a nuclear/lamellar cataract with blue punctate opacities. (**c**) The patient (III3) in Family 3 showed a total cataract. (**d**) The proband (IV12) in Family 5 showed a nuclear cataract

Family 1 had two-generations, including two affected and three unaffected individuals (Fig. 1a). All patients in this family presented bilateral nuclear cataracts with white pupil at birth (Table 2). Nucleus density of lens increased with age presenting a gray opacity, however without other noticeable unusual eye structure. Slit-lamp examination revealed dense nuclear cataract in the center in the II1 (Fig. 2a). All patients were treated using phacoemulsification combined with intraocular lens implantation surgery. The postoperative visual acuity was good and the corrected visual acuity reached 0.5.

**Table 2:** Clinical information of 22 patients from five congenital cataract families

Family number	Patient	Gender	Age at onset	Age at diagnosis (year)	Age at surgery (year)	Phenotype of cataract
Family 1	I2	Female	On birth	9	17	Nuclear, white opacities
	II1	Male	On birth	5	6	Nuclear, white opacities
Family 2	I2	Female	On birth	17	21	Nuclear/lamellar cataract, blue punctate opacities
	II1	Female	On birth	7	9	Nuclear/lamellar cataract, blue punctate opacities
	II2	Female	On birth	2	7	Nuclear/lamellar cataract, blue punctate opacities
Family 3	II3	Female	On birth	20	20	Total cataract
	III1	Female	On birth	2	5	Total cataract
	III3	Female	On birth	2	3	Total cataract
	III4	Female	On birth	1	2	Total cataract
	IV1	Female	On birth	On birth	0.25	Total cataract
Family 4	II1	Male	On birth	10	21	Total cataract
	III2	Female	On birth	2	17	Total cataract
	IV1	Female	On birth	On birth	0.33	Total cataract
	IV2	Male	On birth	On birth	0.17	Total cataract
Family 5	III2	Female	On birth	32	60	Nuclear, white opacities
	IV1	Male	On birth	16	20	Nuclear, white opacities
	IV12	Male	On birth	12	21	Nuclear, white opacities
	V2	Female	On birth	2	5	Nuclear, white opacities
	V12	Female	On birth	On birth	2	Nuclear, white opacities
	V13	Female	On birth	On birth	2	Nuclear, white opacities
	VI1	Male	On birth	On birth	2	Nuclear, white opacities
	VI2	Male	On birth	On birth	2	Nuclear, white opacities

#### Family 2

Five members in a two-generation Chinese family with a history of cataracts participated in the study, including three affected and two unaffected individuals (Fig. 1b). Slit-lamp photograph of proband (II1) showed a nuclear/lamellar cataract with blue punctate opacities (Fig. 2b). All patients with lamellar cataract presented high density of crystal nucleus and crystal density, both of which increased along with age. There was no evidence of other ocular or systemic defects. These features were similar among all the affected participants (Table 2).

#### Family 3

Nine family members of a three-generation Chinese family with a history of cataracts participated in the study, including five affected and four unaffected individuals (Fig. 1c). All patients emerged visible white pupil at birth and crystal gray opacity (Table 2). All patients in this family had bilateral cataracts. The pupils of patient (III3) were white at birth, presented the overall gray crystal (Fig. 2c). While double front sections, intraocular pressures and vitreous body did not show any abnormality.

#### Family 4

Eight family members of a three-generation Chinese family with a history of cataracts participated in the study, including four affected and four unaffected individuals (Fig. 1d). The opacities of proband (IV1) were visible at birth, which had a great influence on visual acuity. She was diagnosed with bilateral total cataract and presented nuclear opacity. These features were similar among all the affected participants (Table 2). There was no evidence of other systemic or ocular defects with the affected family members.

#### Family 5

This family included 9 affected females, 11 affected males and 31 unaffected members in a six-generation pedigree (Fig. 1e). All the affected members in this family were diagnosed as zonular and nuclear cataract coupled with increased crystal nucleus density and they had poor eyesight during the child period of 3~6 months (Table 2). There were no other ocular abnormalities nor other systematic diseases with the patients. The proband (IV12) showed a nuclear cataract (Fig. 2d) and received an operation at age 21 with YAG laser release incision of posterior capsular. Postoperative vision of the proband reached up to 0.6, and vision condition gradually improved after amblyopia training.

### Identification of mutations

**Fig. 3:**
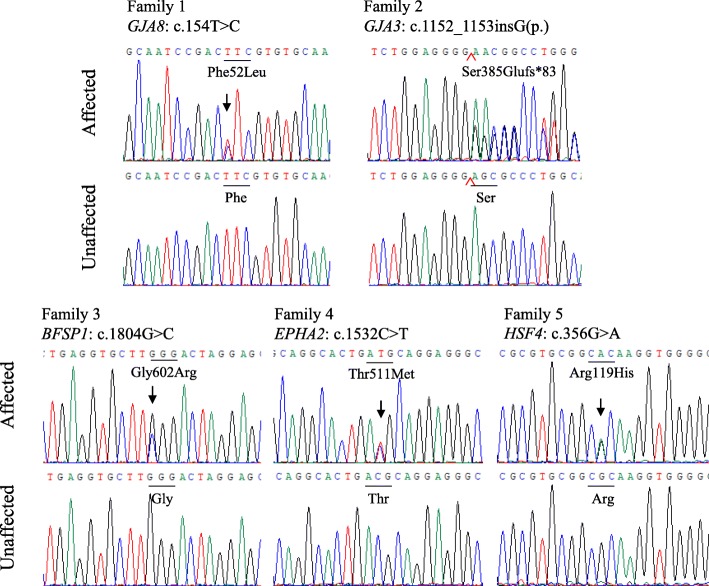
The potential causative mutations were identified in five Chinese families with congenital cataract. (**a**) The heterozygous mutation c.154 T > C(p.F52 L) in *GJA8* was identified in all the affected participants in the Family 1. (**b**) The heterozygous mutation c.1152_1153insG(p.S385Efs*83) in *GJA3* was identified in all the affected participants in the Family 2. (**c**) The heterozygous mutation c.1804G > C(p.G602R) in *BFSP1* was identified in all the affected participants in the Family 3. (**d**) The heterozygous mutation c.1532C > T(p.T511 M) in *EPHA2* was identified in all the affected participants in the Family 4. (**e**) The heterozygous mutation c.356G > A(p.R119H) in *HSF4* was identified in all the affected participants in the Family 5

Thirty-eight genes (Additional file 1: Table S1) related with inheritable and congenital cataract were captured and sequenced by next-generation sequencing. The average coverage was approximately 99.2%, and the average median depth was 475×. 100% of base pairs with N200× coverage was successfully detected indicating high capabilities for identifying variants. Variants in five cataract probands from the targeted NGS in Additional file 2: Table S2. The variants were excluded if they presented high frequency in the 1000 Genome database or the dbSNP database. Since the five family pedigrees accorded with autosomal dominant inheritance, we first focused on heterozygous mutations. Five potential pathogenic mutations were confirmed in the five probands associated with congenital cataract: the heterozygous mutation c.154 T > C (p.F52 L) in *GJA8* in Family 1, c.1152_1153insG (p.S385Efs*83) in *GJA3* in Family 2, and c. 1804 G > C (p.G602R) in *BFSP1* in Family 3, c.1532C > T (p.T511 M) in *EPHA2* in Family 4 and mutation c. 356G > A (p. R119H) in *HSF4* in Family 5. The five mutations were novel and were first identified as associated with congenital cataract. The mutations were further confirmed by Sanger sequencing (Fig. 3), and the five mutations co-segregated with the phenotypes in five families (Fig. 1). Additional testing proved that mutations were not detected in 50 healthy local Chinese controls.

### Bioinformatics analysis of the mutations

**Fig. 4:**
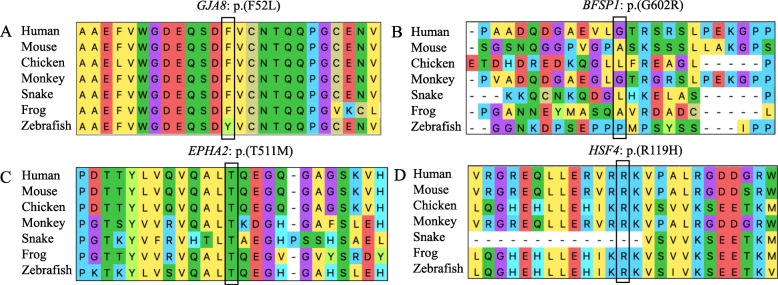
The multiple-sequence alignments from different vertebrate species. (**a-d**) The amino acid alterations, F52 L of *GJA8* in Family 1, T511 M of *EPHA2* in Family 4 and R119H of *HSF4* in Family 5 were located in highly conserved region among all vertebrate species and were marked with box. While G602R of *BFSP1* in Family 3 had lower conservative propertys

**Fig. 5:**
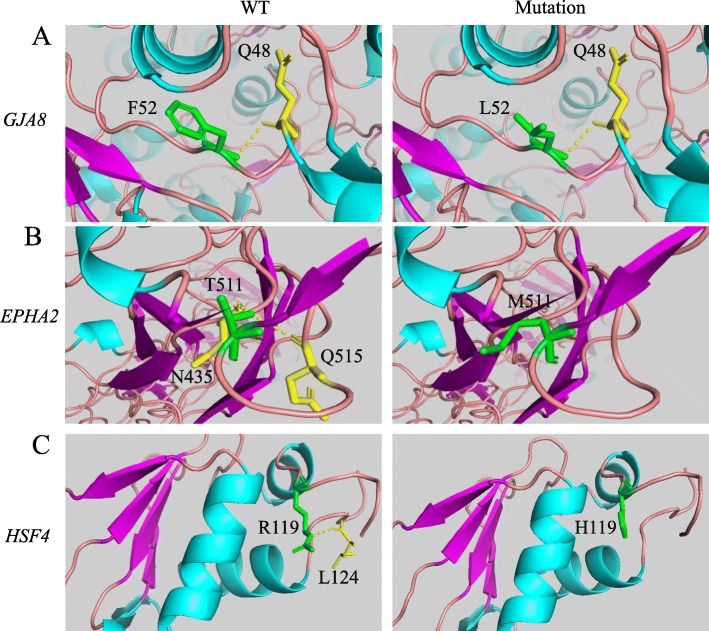
Three missense mutations (F52 L in *GJA8* of Family 1, T511 M in *EPHA2* of Family 4 and R119H in *HSF4* of Family 5) were simulated by means of SWISS-MODEL and were represented with Ribbon model. The proteins were colored by element: a-helix = blue, b-stand = purple, turn = pink. Wild and mutated amino acids were labeled in green. The amino acids that interacted with the mutation sites with hydrogen bonding were marked in yellow. (**a**). Substitution of F52 L in *GJA8* disturbed the core structure domain and influenced the conformation of the protein. (**b**). Substitutions of T511 M in *EPHA2* destroyed the H-bonding between T511 and N435/Q515. (**c**). Substitution of R119H in *HSF4* destroyed the H-bonding between wild-type R119 and L124

Conservation analysis of amino acid located in p.F52 of Gap junction alpha-8, p.G602 of Filensin, p.T511 of Ephrin type-A receptor 2, and p.R119 of Heat shock factor protein 4 within different vertebrate species was performed. The analysis indicated that those p.F52 of Gap junction alpha-8, p.T511 of Ephrin type-A receptor 2, and p.R119 of Heat shock factor protein 4 amino acid sites were highly conserved except p.G602 of Filensin, and the replacement of wild type residues might change their biological function (Fig. 4). In addition, SIFT, PolyPhen-2, MutationTaster, M-CAP and PROVEAN programs yielded similar outcomes regarding pathogenicity except that MutationTaster and PROVEAN predicted p.G602R was a neutral mutation (Table 3). According to the SWISS-MODEL prediction, substitution of phenylalanine into a leucine at position 52 of Gap junction alpha-8 protein would influence the conformation of the protein. In addition, the residue was buried in the core of a domain, and the mutant residue might disturb the core structure of this domain (Fig. 5a). Then, simulation predicted that T511 interacted via H-bonding with residues N435 and Q515 of Ephrin type-A receptor 2. Substitution of M511 destroyed the H-bonding as the original wild-type residue did. (Fig. 5b). Meanwhile, substitution of H119 destroyed the H-bonding, with which wild-type R119 interacted with residues L124 of Heat shock factor protein 4 (Fig. 5c). This indicated that the substitution would affect protein function.

**Table 3:** Pathogenicity prediction of five variants using bioinformatics tools

Family (proband)	Gene	Transcript	Nucleotide change	Amino acid change	SIFT	Polyphen2	MutationTaster	M-CAP	PROVEAN	Pathogenicity (ACMG)	Evidence (ACMG)
Family 1 (II:1)	*GJA8*	NM_005267.4	c.154 T > C	p.F52 L	Deleterious	Possibly damaging	Disease causing	Possibly Pathogenic	Deleterious	Likely Pathogenic	PM1 PM2 PP3 PP4
					0.00	0.478	–	0.513	−5.81	–	–
Family 2 (II:1)	*GJA3*	NM_021954.3	c.1152_1153insG	p.S385Efs*83	Not predicted	Not predicted	Disease causing	Not predicted	Not predicted	Pathogenic	PVS1 PP1 PP4 BP4
Family 3 (IV:1)	*BFSP1*	NM_001195.3	c.1804G > C	p.G602R	Deleterious	Possibly damaging	Polymorphism	Possibly Pathogenic	Neutral	Uncertain significance	PM2 PP1 PP4
					0.02	0.472	–	0.308	−1.03	–	–
Family 4 (IV:1)	*EPHA2*	NM_004431.3	c.1532C > T	p.T511 M	Deleterious	Probably damaging	Disease causing	Possibly Pathogenic	Deleterious	Uncertain significance	PM1 PM2 PP1 PP3 PP4 BS1 BS2 BP6
					0.00	1.00	–	0.04	−4.80	–	–
Family 5 (IV:12)	*HSF4*	NM_001538.3	c. 356G > A	p. R119H	Deleterious	Probably damaging	Disease causing	Possibly Pathogenic	Deleterious	Pathogenic	PM1 PM2 PP1 PP2 PP3 PP4 BP1
					0.00	1.00	–	0.259	−4.81	–	–

## Discussion

We reported five novel mutations associated with the autosomal dominance cataract in five Chinese families respectively: c.154 T > C in *GJA8*, c.1152_1153insG in *GJA3*, c.1804G > C in *BFSP1*, c.1532C > T in *EPHA2* and c.356G > A in *HSF4*. All of the five mutations were screened by targeted NGS for the 38 candidate genes of congenital cataracts, and verified through Sanger DNA sequencing. We confirmed that each mutation co-segregated with the disease phenotypes in the corresponding family and absent in all the unaffected individuals. Further, bioinformatics analysis, conservative prediction and 3-D protein simulation showed that these mutations might be deleterious. According to the ACMG criteria [20], c.1152_1153insG in *GJA3* of Family 2 and c.356G > A in *HSF4* of Family 5 are clearly pathogenic variants (class V); c.154 T > C in *GJA8* of Family 1 is a likely pathogenic variant (class IV); c.1804G > C in *BFSP1* of Family 3 and c.1532C > T in *EPHA2* of Family 4 variants are unknown significance (class III) (Table 3). The unknown significance variants associated with congenital cataracts make them interesting candidates for further studies.

The lens has developed an extensive cell-cell interaction system using connexins to maintain its transparency. Three connexins are expressed in the lens: connexin 43 (Cx43), connexin 46 (Cx46), and connexin 50 (Cx50). Cx43 (*GJA1*) is expressed mainly in epithelial cells of lens, while Cx46 (*GJA3*) and Cx50 (*GJA8*) are expressed in lens fibre cells [21, 22]. *GJA8* and *GJA3* are the major connexin of the ocular lens, where gap junctions maintain ionic environment, water balance, transparency and optical properties of the lens [23]. To date, 65 variants in *GJA8* and 43 variants in *GJA3* have been reported in the HGMD (Professional 2019.1) to induce genetic cataracts, which account for about 1/4 of nonsyndromic familial cataract cases. The typical structure of connexin includes cytoplasmic NH2- and COOH- terminal domain, four transmembrane domains and two extracellular loops. The two extracellular loops mediate hemichannel docking between connexons and the E1 loop, which was also shown to be important for the voltage required for closure of gap junction pores [24]. In this study, we identified an amino acid change (F52 L) at the first external loop (E1) in *GJA8* in family 1. The altered protein may disrupt normal interactions between the two connexins, which may reduce resistance of the intercellular channel and lead to the leakage of small ions. Moreover, F52 L is highly conserved among many species, so F52 L is very likely to cause disease. In Family 2, frameshift S385Efs*83 in *GJA3* resulted from a guanine insertion that introduced a premature translation stop codon located in the COOH-terminus, which may interfere with the folding of the whole protein and resulted in cataract. This insertion mutation (c.1152_1153insG) is similar to the three mutations (c.1137dupC, c.1189dupG, c.1200dupC) reported previously [25–27], thus providing further evidence that the *GJA3* C-terminal domain plays an essential role in physiological function of the gene, and further expanding the mutation spectrum of *GJA3* in association with congenital cataract.

BFSP1 (filesin) and BFSP2 (phakinin) are major components of the beaded filament, which are unique cytoskeletal lens structures. The biological functions of filesin and phakinin are still not clear, but some evidences indicate they play an important role in maintaining lens transparency and homeostasis during fetal development and fiber cell differentiation [28]. A novel mutation c.1804G > C(p.G602R) in *BFSP1* was detected in Family 3. Alignment of the BFSP1 protein sequence among different species revealed that the Gly residue at position 602 was less conservative. MutationTaster and PROVEAN prediction tools showed the pathogenicity of G602R was neutral. However, M-CAP, SIFT and PolyPhen 2 analysis indicated that G602R was possibly damaging. Further, mutation was co-segregated with phenotypes in the Family 3 including five affected and four unaffected individuals and that variant frequency was 0.000066 in the ExAC browser, indicating that this variant was rare event in the human genome. Up to now, only six *BFSP1* mutations have been reported and four *BFSP1* mutations were involved in autosomal recessive cataract families [11, 29, 30]. And two mutations were found in autosomal dominant cataract families. In 2013, Wang et al. first found a heterozygous variant c.1042G > A(p.D348N) in *BFSP1* in a 5-generation Chinese family in which 15 members had autosomal dominant nuclear cataract [31]. In 2017, Zhai et al. identified heterozygosity for a splice site mutation (c.625 + 3A > G) in *BFSP1* in a 4-generation family co-segregating progressive punctate lamellar cataract [32]. The mutation (G602R) highlighted in this study is localized at the tail region of filesin, has an important effect on beaded filament formation as mutation D348N [31]. Taken together with previous research, the results of the Family 3 enriched the suspected pathogenicity of the *BFSP1* mutation in human autosomal dominant congenital cataract.

The protein encoded by *EPHA2,* Ephrin Receptor EphA2, is spatially and temporally regulated in the cortical lens fiber cells, while its expression is lower in anterior epithelial cells, and absent in the nuclei of lens [33]. So far, 22 mutations of *EPHA2* have been reported in the patients with congenital cataract, and most of them are in the SAM domain. After identification of p.P584L by Dave et al., we reported the second autosomal dominant mutation p.T511 M in the juxta membrane domain of the protein [34]. The pathogenicity of this mutation was proved in the following three aspects: (1) Protein sequence among different species revealed that the Thr residue at position 511 was highly conserved; (2) Bioinformatics analysis using five prediction tools indicated that T511 M was a pathogenic change. (3) 3-D protein simulation model predicted that amino acids change of M511 T would destroy H-bonding between T511 interacted via with residues N435 and Q515 of Ephrin type-A receptor 2. Furthermore, this mutation was co-segregated with phenotypes in Family 4. The mutation T511 M identified in *EPHA2* gene is a known polymorphism (rs55747232), which raises doubt about its pathogenicity. In conclusion, we believe that M511 T in *EPHA2* is a potential variation associated with congenital cataract.

*HSF4* belongs to the family of heat-shock transcription factors that bind heat shock elements and activate downstream heat-shock response genes under conditions of stress [35]. It has been reported that *HSF4* gene is responsible for both autosomal dominant and autosomal recessive cataracts [36]. We had screened the affected individuals in Family 5 and identified a missense mutation c.356G > A(p.R119H) in *HSF4,* and this mutation was co-segregated with the disease in all the affected individuals, but not observed in all the unaffected individuals. Protein sequence among different species revealed that the Arginine(R) residue at position 119 is high conserved, and five prediction tools showed p.R119H is pathogenic. 3-D protein simulation predicted that substitution of H119 destroyed the H-bonding, with which wild-type R119 interacted with residues L124 of Heat shock factor protein 4. Above all provided a persuasive evidence to its pathogenicity of p.R119H in *HSF4* of Family 5*.*

In summary, we performed genetic analysis in five Chinese families with congenital dominant cataracts and identified five novel mutations, including an insertion mutation encoding p.S385Efs*83 in *GJA3* and four missense mutations: p.F52 L in *GJA8*, p.G602R in *BFSP1*, p.T511 M in *EPHA2* and p.R119H in *HSF4*. This work extended the mutation spectrum of congenital cataracts, and would provide more evidences for the precise diagnosis of the disease.

## Conclusions

We firstly reported five novel mutations associated with autosomal dominant cataracts: c.154 T > C in *GJA8*, c.1152_1153insG in *GJA3*, c.1804G > C in *BFSP1*, c.1532C > T in *EPHA2*, c.356G > A in *HSF4*. This study expands the mutation spectrum of congenital cataracts, and provide solid evidence for genetic counseling and prenatal gene diagnosis of the cataract families.

## Supplementary information

**Additional file 1: Table S1.**. Information of 38 candidate genes for congenital cataract used in targeted NGS.

**Additional file 2: Table S2.** Variants in five cataract probands from the targeted NGS.

## Data Availability

The datasets generated and/or analysed during the current study are available in the CNGB Nucleotide Sequence Archive (CNSA: https://db.cngb.org/cnsa; accession number CNP0000764).

## Abbreviations

BFSP1: Beaded filament structural protein 1
EPHA2: EPH receptor A2
GJA3: Gap junction protein alpha 3
GJA8: Gap junction protein alpha 8
HGMD: Human Gene Mutation Database
HSF4: Heat shock transcription factor 4
M-CAP: Mendelian Clinically Applicable Pathogenicity
NGS: Next generation sequencing
PCR: Polymerase chain reaction
PolyPhen-2: Polymorphism Phenotyping v2
PROVEAN: Protein variation effect analyzer
SIFT: Scale-Invariant Feature Transform
UCSC: University of California, Santa Cruz
